# Acidic domains: “converse readers” for acetylation code

**DOI:** 10.18632/oncotarget.12989

**Published:** 2016-10-29

**Authors:** Donglai Wang, Ning Kon, Wei Gu

**Affiliations:** Department of Pathology and Cell Biology, Institute for Cancer Genetics, Herbert Irving Comprehensive Cancer Center, College of Physicians & Surgeons, Columbia University, New York, NY, USA

**Keywords:** p53, SET, acetylation, acidic domain-containing protein, gene-specific transcription

During past decades, acetylation has emerged as a general post-translational modification that is widespread and distributed on lysine residues of histones and non-histone proteins. Lysine acetylation has been suggested to create a platform for the recruitment of bromodomain-containing proteins that serve as “readers” to decode information within the acetylated lysine residues [1]. However, the precise mechanism of acetylation-mediated regulatory effects is still not fully understood. p53 is the first identified non-histone protein modified by lysine acetylation; p53 acetylation regulates p53-dependent gene-specific transcription during stress responses [2]. Our recent study focusing on acetylation of the p53 C-terminal domain (CTD) revealed the acidic domain-containing proteins as novel “converse readers” of protein acetylation [3]. Unlike bromodomain “readers” that preferentially bind with acetylated ligands, the acidic domain “readers” specifically recognize the unacetylated forms of their ligands.

By performing an *in vitro* proteomic screen, we identified the oncoprotein SET as a major binding partner of p53 when the CTD is unacetylated; conversely, this p53-SET interaction was completely abolished by CTD acetylation. Of note, this acetylation-mediated regulation was unique, as other modifications on the same lysine residues including methylation, ubiquitination, sumoylation and neddylation, had no obvious effect on p53-SET interaction. Functionally, SET negatively regulated p53 transactivity by acting as a co-repressor inhibiting p300/CBP-mediated H3K18 and H3K27 acetylation on p53 target promoters. Depletion of endogenous SET in p53-wildtype cells significantly suppressed xenograft tumor formation, indicating that p53-SET interplay is crucial for p53-mediated tumor growth repression. During DNA damage response, p53 is stabilized and CTD acetylation is highly induced; the p53-SET interaction in both soluble and chromatin-bound fractions was severely disrupted, releasing p53 in an activated status. This observation was further proved in physiological conditions by using a CTD acetylation-mimicking mouse model (*p53KQ/KQ*) where p53 failed to interact with SET. More importantly, *p53KQ/KQ* mice exhibited neonatal lethality due to p53 hyperactivity in multiple tissues, consistent with the notion that CTD acetylation activates p53.

SET consists of an N-terminal dimerization domain, a middle “earmuff” domain and a C-terminal acidic domain (AD). Biochemical analysis indicated that the p53 CTD directly interacted with the SET AD. The p53 CTD is a lysine-rich domain, which harbors a positive charge. Conversely, the SET AD is enriched with acidic amino acids, exhibiting a highly negative charge. Therefore, we postulated that p53-SET interaction is mediated by a charge effect between the CTD and the AD. Upon acetylation, the positive charge of the CTD is neutralized, resulting in a dissociation of the p53-SET complex due to disruption of the charge effect between the CTD and the AD. Bioinformatic analysis of human proteomics revealed that numerous proteins contain acidic domains or lysine-rich domains; our additional binding assays suggested a general mechanism that the acidic domain acts as a “reader” of unacetylated lysine residues and acetylation acts as a “switch” to determine the interaction between acidic domain-containing proteins and their lysine-rich domain-containing ligands.

This mechanism was further illustrated by the observation that other acidic domain-containing factors of p53 including VPRBP, DAXX and PELP1, also interacted with the p53 CTD through their acidic domains; of note, their interactions were completely blocked by CTD acetylation. More importantly, all these proteins lost their interactions with p53 in *p53KQ/KQ* mouse model, demonstrating the property of CTD acetylation-mediated dissociation of p53 from its acidic domain-containing factors *in vivo*.

**Figure 1:**
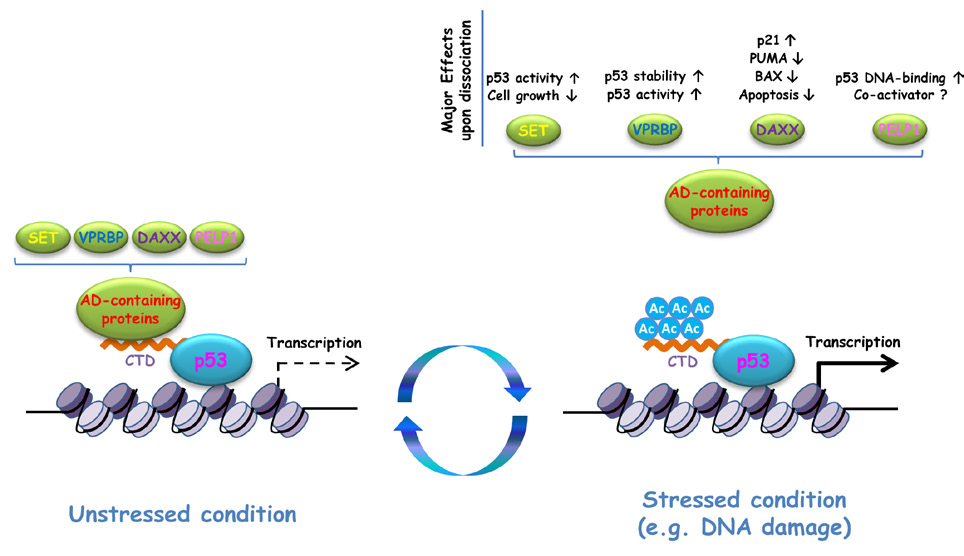
Schematic diagram of C-terminal domain (CTD) acetylation-mediated regulation of p53- dependent transcription In unstressed conditions, unacetylated p53 recruits various acidic domain (AD)- containing proteins (e.g. SET, VPRBP, DAXX and PELP1) due to the charge effect between the p53 CTD and the acidic domain of these proteins. Upon stresses (e.g. DNA damage), the p53 CTD acetylation is highly induced, which in turn neutralizes the positive charge of the CTD and abolishes p53 interactions with these AD-containing proteins. AD-containing proteins, in a separate or a combined manner, modulate p53 in multiple aspects (e.g. stability, DNA-binding affinity), which may contribute to gene-specific transcription of p53 downstream targets.

Based on these observations, a question is raised: why do many acidic domain-containing factors “read” p53Œ One explanation is that the context-dependent association/dissociation of distinct acidic domain-containing factors to p53 contributes to fine-tune the regulation of p53 stability, DNA-binding affinity and gene-specific transcription of p53 downstream targets (Fig. 1). For example, recruitment of SET represses multiple p53 targets (tending to inhibit p53-dependent cell growth arrest) without affecting p53 DNA-binding affinity or stability. In contrast, VPRBP is involved in p53 degradation and transcriptional repression through H2A phosphorylation [4, 5]. As another example, DAXX represses p21, the major p53-downstream target regulating the cell cycle, but facilitates the expression of pro-apoptotic targets PUMA and BAX [6]. PELP1 influences p53 DNA-binding affinity and optimizes p53-dependent transcription of both cell cycle arrest and pro-apoptotic targets [7]. In unstressed condition, these factors may separately or synergistically interact with p53, or compete with each other to interact with p53 due to differences in binding affinity of their acidic domain to p53 CTD. In response to various stresses, p53 CTD acetylation is dynamically induced into different levels (partially vs. completely) that could determine which groups of interactions between acidic domain-containing factors and p53 are kept or abolished in a given situation. This specific acidic domain binding regulation may determine how p53-dependent transcription patterns are established and regulated. Although future investigations are clearly required, it is very likely that these different “readers” play crucial roles in promoter-specific regulation of *in vivo* targets of p53.
